# Assessing female sexual dysfunction in patients with relapsing-remitting multiple sclerosis

**DOI:** 10.1055/s-0045-1806819

**Published:** 2025-04-22

**Authors:** Elisa Matias Vieira de Melo, Flavia Fairbanks Lima de Oliveira Ruano, Maria Fernanda Mendes, Guilherme Sciascia do Olival

**Affiliations:** 1 Santa Casa de São Paulo, Faculdade de Ciências Médicas, São Paulo SP, Brazil.; 2 University of Miami, Department of Obstetrics Gynecology and Reproductive Sciences, Female Sexual Health Program, Coral Gables FL, United States.

**Keywords:** Multiple Sclerosis, Sexual Health, Sexual Dysfunction

## Abstract

**Background** Multiple sclerosis (MS) affects mainly young people of reproductive age with significant lifelong repercussions, among which sexual dysfunction (SD) is one of the most neglected during routine clinical care.

**Objective** To evaluate SD in female patients diagnosed with relapsing-remitting MS (RRMS).

**Methods** This cross-sectional analytic study was performed at the Santa Casa de São Paulo Hospital, Faculdade de Ciências Médicas, São Paulo, Brazil, between November 2020 and March 2022. The sample included 80 female patients diagnosed with RRMS and 106 healthy controls. Questionnaires probing sexual dysfunction (the Multiple Sclerosis Intimacy and Sexuality Questionnaire – MSISQ-19 and the Female Sexual Function Index – FSFI) and depression and anxiety (the Hospital Anxiety and Depression Scale – HADS, the Beck Depression Inventory – BDI; and the Beck Anxiety Inventory – BAI) were applied.

**Results** A high prevalence of SD in both groups (43.4% and 38.8% for the RRMS and control groups, respectively) was identified by the FSFI analysis. A statistically higher prevalence (56.3%) of sexual dysfunction was detected in RRMS patients when using the MSISQ-19 tool to assess sexuality in this population compared with the FSFI scale ( *p* = 0.016).

**Conclusion** A high prevalence of SD was found in both MS patients and healthy controls as measured by the FSFI. However, the specific tool (MSISQ-19) revealed a higher prevalence of SD in MS patients. Thus, the use of MSISQ-19 for the diagnosis and management of SD in this patient group is recommended.

## INTRODUCTION

Multiple sclerosis (MS) is a chronic disease of the central nervous system (CNS). 1 2 The age of onset is typically between 15 and 45 years, and women are 3 times more likely to be affected than men; 3 therefore, it has a disproportionate impact on reproductive-aged women.

Improvements in early diagnosis and the development of disease-modifying therapies (DMTs) have led to reduced levels of disability and disease progression. 1 However, patients with MS still have unmet needs that have a significant impact on health, wellness, and quality of life. Factors such as fatigue, cognitive deficit, sleep disturbances, mood disorders, and sexual dysfunction often remain overlooked. 4 5 6 7

Sexual dysfunctions (SDs) can be broadly described as a range of issues that prevent individuals from experiencing satisfaction from sexual activity, manifesting in a persistent or recurrent manner. According to the World Health Organization (WHO), SDs can involve disturbances in sexual desire, the ability to achieve or maintain an erection, lubrication, orgasm, or experiencing pain during intercourse. 8 According to the Diagnostic and Statistical Manual of Mental Disorders, 5th edition, a diagnosis of SD requires a person to feel extreme distress and interpersonal strain for a minimum of 6 months (except for substance- or medication-induced sexual dysfunction). 9

**Figure 1:**
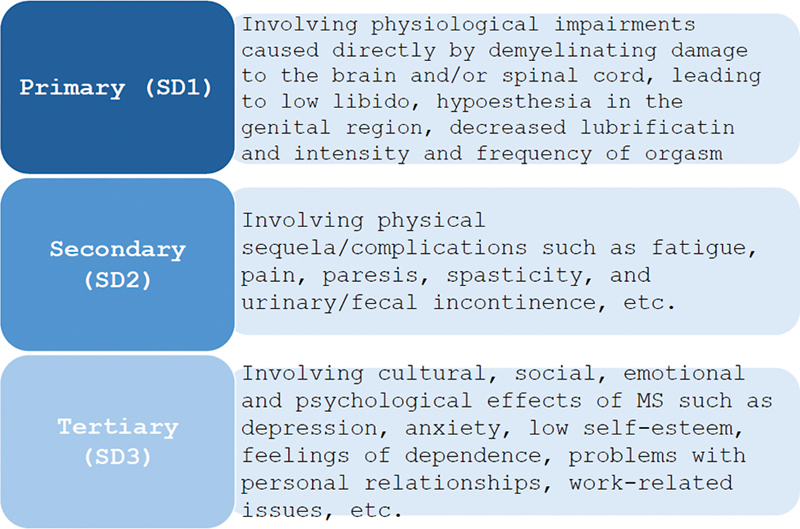
Model of sexual dysfunction categories in people with multiple sclerosis. Adapted from Foley and Iverson, 1992. ^10^

Sexual dysfunction in patients with MS is multifactorial and may be influenced by physical, organic, hormonal, and psychological factors. Foley and Iverson defined a conceptual model with three forms of SD in MS ( Figure 1 ): primary (SD1) involving physiological impairments caused directly by demyelinating damage to the brain and/or spinal cord, leading to low libido, hypoesthesia in the genital area, decreased lubrication and intensity and frequency of orgasm; secondary (SD2) involving physical symptoms such as fatigue, decreased mobility, and fecal/urinary incontinence; and tertiary (SD3) involving the cultural, social, emotional, and psychological effects of MS. 10

Although the prevalence of SD in patients with MS has been estimated to be 40 to 80%, patients rarely discuss their sexual health with their providers. 11 12 13 In a study of 137 female patients with MS, Lew-Starowicz and Rola (2013) found that only 2.2% were asked about their sexual health by their providers. 14 Petracca et al. (2023) found that only 18% of patients with MS reported discussing their sexual health with their treating neurologists. 15 These studies indicate that there has been negligible progress in respect of healthcare professionals discussing this topic with their patients.

Sexual dysfunction can be evaluated using validated questionnaires, such as the Female Sexual Function Index (FSFI) for the general population and the MS Intimacy and Sexuality Questionnaire-19 (MSISQ19), a specific scale for assessing people diagnosed with MS. 16 17 18 19 20

The MSISQ-19 is a self-administered 19-item SD assessment scale for measuring the influence of MS symptoms on sexual activity and satisfaction, evaluating their impact on the patient's sex life in the 6 months prior to the application of the questionnaire. The instrument is divided into 3 subscales: SD1, for primary SD (items 12, 16, 17, 18, and 19); SD2, secondary (items 1, 2, 3, 4, 5, 6, 8, 10, and 11), and SD3, tertiary (items 7, 9, 13, 14, and 15). Each item is rated from 1 to 5 (1 = never; 2 = seldom; 3 = sometimes; 4 = often, and 5 = always), and any item scoring 4 or 5 should be discussed with your MS health care provider. 16 17 18

The Female Sexual Function Index (FSFI) is considered the gold standard for assessing female sexual function, with over 1,000 articles citing its use, and at least 20 versions translated into other languages with confirmed validity and reproducibility. The index is relatively quick to apply (average time ≤ 5 minutes) and has a practical, direct scoring system. It includes 19 questions that evaluate 6 sexual domains (desire, arousal, lubrication, orgasm, satisfaction, and pain). A score below 26.55 indicates the presence of SD. 19 20

Despite the high prevalence of SD in this population and its significant impact on quality of life, the sexuality of patients with MS is seldom explored during routine clinical visits. The goal of the present study is to identify the prevalence of SD in female patients with MS and to analyze whether there is a higher prevalence in this cohort compared with the general population. Additionally, the study aims to assess if there is a difference in prevalence according to the assessment tool used: a tool specific to this population (MSISQ-19) versus the gold standard for the general population (FSFI).

## METHODS

Patients with relapsing-remitting multiple sclerosis (RRMS) and healthy controls were enrolled in a cross-sectional survey study at the School of Medical Sciences of Irmandade da Santa Casa de Misericórdia de São Paulo, São Paulo, Brazil, from November 2020 to March 2022.

Patients diagnosed with RRMS were recruited from our neuroimmunology service during their consultations. Women in the control group were recruited during routine visits with a general practitioner and gynecologist in primary/basic healthcare, with demographic characteristics similar to those of the patients who attended our service.

A total of 80 consecutive female patients were included in the RRMS group. The inclusion criteria for the MS group were: RRMS diagnosis according to McDonald's criteria, age 18 to 45 years, female gender, and the ability to complete the questionnaire without assistance. The exclusion criteria were the absence of sexual activity in the last 6 months, menopause/postmenopause/premature ovarian failure, current pregnancy, postpartum status or breastfeeding, and failure to fully complete the following assessment tools: the FSFI, the MSISQ-19, the Hospital Anxiety and Depression Scale (HADS), the Beck Depression Inventory (BDI), and the Beck Anxiety Inventory (BAI). All patients responded to the questionnaires and underwent a neurological examination by the same neurologist, including the following functional tests to assess functionality and sequelae related to the underlying disease: the Expanded Disability Status Scale (EDSS), the Nine-Hole Peg Test (9HPT), and the Timed 25-Foot Walk (T25FW).

Of the 116 questionnaires completed by the control group, 106 were validated for data assessment. Ten control subjects were excluded for not meeting the study criteria. The inclusion criteria for the control group were: age 18 to 45 years, female gender, no diagnosed neurological disease at the time of the interview, and the ability to complete the questionnaire without assistance. The exclusion criteria were the absence of sexual activity in the last 6 months, menopause/postmenopause/premature ovarian failure, being pregnant, in postpartum or nursing, and not fully completing the assessment tools (FSFI, MSISQ-19, HADS, BDI, and BAI).

Demographic data were collected from all participants. Baseline characteristics were expressed as proportions and mean ± SD or median and interquartile range (IQR) values, according to data distribution. The presence of SD was described according to the qualitative parameters evaluated, and associations were determined using the Chi-squared or exact test. Quantitative characteristics were compared with the presence of dysfunction using the Student's *t* -test or the Mann-Whitney test. Statistical analyses were performed using the IBM SPSS Statistics for Windows (IBM Corp., Armonk, NY, USA) software, version 20.0.

All participants signed a Free and Informed Consent Form. The ethics committee of Irmandade da Santa Casa de Misericórdia de São Paulo approved this research project for research in humans under permit number 4.130.410 on July 2, 2020.

## RESULTS

The characteristics presented in Table 1 reveal the homogeneity of the groups in terms of age, ethnicity, years of schooling, body mass index (BMI), occupational status, and family income. There was a statistically significant group difference for marital status, with a higher proportion of married patients in the RRMS patient group. The clinical characteristics assessed in patients with RRMS are presented in Table 2 .

**Table 1:** Descriptive characteristics of the study sample

Variable		Group		*p*
		Control ( *N* = 106)	RRMS ( *N* = 80)
**Age (years): mean ± standard deviation**		32.3 ± 8	33.8 ± 6.5	0.146
**Self-declared ethnicity, n (%)**	White	66 (62.3)	56 (70)	**0.044**
	Black	39 (36.8)	24 (30)	
	No answer	1 (0.9)	0 (0)	
**Relationship status, n (%)**	Steady relationship	53 (50)	58 (72.5)	**0.002**
	No steady relationship	53 (50)	22 (27.5)	
		**(** ***N*** ** = 83)**	**(** ***N*** ** = 80)**	
**Years of schooling:** **n (%)**	0–8	1 (1.2)	2 (2.5)	0.501
	9–11	42 (50.6)	39 (48.8)	
	> 12	40 (48.2)	39 (48.8)	
		**(** ***N*** ** = 102)**	**(** ***N*** ** = 80)**	
** BMI (Kg/m ^2^ ): ** **mean ± standard deviation**		25.7 ± 4.3	27 ± 6.3	0.097
		**(** ***N*** ** = 104)**	**(** ***N*** ** = 78)**	
**Occupational status: n (%)**	Employed	77 (74)	50 (64.1)	0.149
	Unemployed	27 (26)	28 (35.9)	
		**(** ***N*** ** = 101)**	**(** ***N*** ** = 65)**	
**Family income in BRL reais/per month** : **n (%)**	> 22,000	9 (8.9)	1 (1.5)	0.496
	11,000–22,000	17 (16.8)	9 (13.8)	
	4,400–11,000	29 (28.7)	26 (40)	
	2,200–4,400	30 (29.7)	22 (33.8)	
	< 2,200	16 (15.8)	7 (10.8)	

Abbreviations: BMI, body mass index; RRMS, Relapsing-Remitting Multiple Sclerosis.

**Table 2:** Description of the clinical characteristics of RRMS patients

Variable		Value ( *N* = 80)
		( *N* = 79)
**Age at symptom onset (years): mean ± standard deviation**		24.9 ± 6.8
		**(** ***N*** ** = 79)**
**Age at diagnosis (years): mean ± standard deviation**		26.4 ± 6.8
		**(** ***N*** ** = 80)**
**Disease duration (years): mean ± standard deviation**		7.7 ± 6.6
		**(** ***N*** ** = 80)**
**Number of drugs used: mean ± standard deviation**		0.97 ± 0.95
		**(** ***N*** ** = 80)**
**EDSS: mean ± standard deviation**		2.26 ± 1.81
		**(** ***N*** ** = 78)**
**Mean T25FW (seconds): mean ± standard deviation**		6.6 ± 4.4
		**(** ***N*** ** = 79)**
**Mean 9HPT (seconds): mean ± standard deviation**		25.3 ± 6.3
		**(** ***N*** ** = 80)**
**Current medication: n (%)**	First line	15 (18.8)
	Second line	29 (36.3)
	Third line	30 (37.5)
	No medication	6 (7.5)

Abbreviations: EDSS, Expanded Disability Status Scale; 9HPT, Nine-Hole Peg Test (9HPT); RRMS, Relapsing-Remitting Multiple Sclerosis; T25FW, Timed 25-Foot Walk.

Notes: First line – interferon, glatiramer acetate, and teriflunomide; second line – Dimethyl fumarate and Fingolimod; third line: cladribine, natalizumab, ocrelizumab, rituximab, alemtuzumab.

The prevalence of sexual dysfunction measured by the FSFI was 38.8% in the RRMS group versus 43.4% in the control group, with the difference between the two groups not being statistically significant. Moreover, a comparison of the FSFI domain scores of patients with sexual dysfunction (FSFI < 26.55) in the RRMS group versus the control group revealed no statistically significant difference ( *p* > 0.05).

The prevalence of general SD in patients with RRMS measured by the MSISQ-19 was 56.3% ( Table 3 ). Secondary SD was the most prevalent (42.5%) category in the patients with RRMS, followed by SD1 and SD3 ( Table 4 ).

**Table 3:** Description of the prevalence of sexual dysfunction, overall and on the MSISQ-19 subscales, in the RRMS patient group

Variable		Value ( *N* = 80)
**MSISQ-19: n (%)**	Yes	45 (56.3)
	No	35 (43.8)
**SD1: n (%)**	Yes	29 (36.3)
	No	51 (63.7)
**SD2: n (%)**	Yes	34 (42.5)
	No	46 (57.5)
**SD3: n (%)**	Yes	27 (33.8)
	No	53 (66.3)

Abbreviations: MSISQ-19, Multiple Sclerosis Intimacy and Sexuality Questionnaire-19; SD, sexual dysfunction; SD1, primary sexual disfunction; SD2, secondary sexual disfunction; SD3, tertiary sexual disfunction.

**Table 4:** Frequency of distribution and mean of symptoms assessed by subscale for primary, secondary, and tertiary sexual dysfunction in the RRMS patient group according to the MSISQ-19

SD subscale	Never: N (%)	Rarely: N (%)	Occasionally: N (%)	Almost always: N (%)	Always: N (%)	Mean(± standard deviation)	SD:* n (%)
**SD1**							
12. Less feeling or numbness in my genitals	47 (58.8)	12 (15.0)	10 (12.5)	7 (8.8)	4 (5.0)	1.86(± 1.23)	11 (13.8)
16. Lack of sexual interest or desire	33 (41.3)	12 (15.0)	19 (23.8)	10 (12.5)	6 (7.5)	2.3(± 1.33)	16 (20.0)
17. Less intense or pleasurable orgasms or climaxes	36 (45)	18 (22.5)	15 (18.8)	9 (11.3)	2 (2.5)	2.16(± 1.52)	11 (13.8)
18. Takes too long to orgasm or climax	27 (33.8)	17 (21.3)	17 (21.3)	14 (17.5)	5 (6.3)	2.41(± 1.29)	19 (23.8)
19. Inadequate vaginal wetness or lubrication (women)/difficulty getting or keeping a satisfactory erection (men)	46 (57.5)	10 (12.5)	10 (12.5)	12 (15)	2 (2.5)	1.92(± 1.24)	14 (17.5)
**SD2**							
1. Muscle tightness or spasm in my arms, legs, or body	43 (53.8)	19 (23.8)	6 (7.5)	9 (11.3)	3 (3.8)	1.88(± 1.18)	12 (15)
2. Bladder or urinary symptoms	47 (58.8)	12 (15)	17 (21.3)	2 (2.5)	2 (2.5)	1.75(± 1.04)	4 (5)
3. Bowel symptoms	49 (61.3)	12 (15)	15 (18.8)	2 (2.5)	2 (2.5)	1.7(± 1.02)	4 (5)
4. Feelings of dependency because of MS	47 (58.8)	10 (12.5)	16 (20)	2 (2.5)	5 (6.3)	1.85(± 1.20)	7 (8.8)
5. Tremors or shaking in my hands or body	40 (50)	20 (25)	15 (18.8)	1 (1.3)	4 (5)	1.86(± 1.09)	5 (6.3)
6. Pain, burning, or discomfort in my body	45 (56.3)	12 (15)	12 (15)	5 (6.3)	6 (7.5)	1.94(± 1.29)	11 (13.8)
8. Problems moving my body the way I want during sexual activity	42 (52.5)	14 (17.5)	13 (16.3)	6 (7.5)	5 (6.3)	1.96(± 1.25)	11 (13.8)
10. Problems with concentration, memory, or thinking	24 (30)	14 (17.5)	17 (21.3)	18 (22.5)	7 (8.8)	2.62(± 1.35)	25 (31.3)
11. Exacerbation or significant worsening of my MS	59 (73.8)	10 (12.5)	6 (7.5)	4 (5)	1 (1.3)	1.48(± 0.93)	5 (6.3)
**SD3**							
7. Feeling that my body is less attractive	31 (38.8)	13 (16.3)	14 (17.5)	15 (18.8)	7 (8.8)	2.43(± 1.39)	22 (27.5)
9. Feeling less masculine or feminine due to MS	54 (67.5)	9 (11.3)	11 (13.8)	5 (6.3)	1 (1.3)	1.63(± 1.02)	6 (7.5)
13. Fear of being rejected sexually because of MS	57 (71.3)	8 (10)	6 (7.5)	8 (10)	1 (1.3)	1.6(± 1.07)	9 (11.3)
14. Worries about sexually satisfying my partner	41 (51.3)	9 (11.3)	19 (23.8)	6 (7.5)	5 (6.3)	2.06(± 1.28)	11 (13.8)
15. Feeling less confident about my sexuality due to MS	50 (62.5)	12 (15)	9 (11.3)	5 (6.3)	4 (5)	1.76(± 1.18)	9 (11.3)

Abbreviations: MS, multiple sclerosis; MSISQ-19, Multiple Sclerosis Intimacy and Sexuality Questionnaire-19; RRMS, Relapsing-Remitting Multiple Sclerosis; SD, sexual dysfunction; SD1, primary sexual disfunction; SD2, secondary sexual disfunction; SD3, tertiary sexual disfunction.

Analysis of the subdomains revealed that for each additional year of disease duration, the odds of SD2 decreased by 12% (odds ratio [OR] = 0.88 (0.78–1.00); *p* = 0.041). A high BMI had a statistically significant influence on the prevalence of SD3 ( *p* = 0.029).

The prevalence of general SD in patients with MS according to the MSISQ-19 scale was statistically higher compared with the results of the FSFI scale ( *p* = 0.016). ( Table 5 ).

**Table 5:** Comparison of sexual dysfunction prevalence on the MSISQ-19 and FSFI scales in the RRMS patient group

Variable: n (%)		FSFI qualitative		Total	*p*
		No	Yes		
**MSISQ-19 qualitative**	No	27 (33.8)	8 (10)	35 (43.8)	**0.016**
	Yes	22 (27.5)	23 (28.7)	45 (56.3)	
**SD1**	No	38 (47.5)	13 (16.3)	51 (63.7)	0.839
	Yes	11 (13.8)	18 (22.5)	29 (36.3)	
**SD2**	No	32 (40)	14 (17.5)	46 (57.5)	0.720
	Yes	17 (21.3)	17 (21.3)	34 (42.5)	
**SD3**	No	36 (45)	17 (21.3)	53 (66.3)	0.585
	Yes	13 (16.3)	14 (17.5)	27 (33.8)	
**Total**		**49** **(61.3)**	**31 (38.8)**	**80 (100)**	

Abbreviations: FSFI, Female Sexual Function Index; MSISQ-19, Multiple Sclerosis Intimacy and Sexuality Questionnaire-19; RRMS, Relapsing-Remitting Multiple Sclerosis; SD1, primary sexual dysfunction; SD2, secondary sexual dysfunction; SD3, tertiary sexual dysfunction.

The prevalence of anxiety and depression was similar between the groups, with 50% and 42% anxiety and 40% and 29% depression, respectively, in the control and patient groups, according to the Beck scale ( *p* > 0.05). However, SD1 was more frequent in patients with RRMS who exhibited symptoms of depression according to the HADS and BDI scales ( *p* = 0.033 and *p* = 0.011, respectively) and with symptoms of anxiety according to the BAI scale ( *p* = 0.032), with statistically significant differences.

In the analysis of independent factors for SD in patients with RRMS, regarding the duration of the disease, each additional year of the disease decreased the chance of SD2 by 12%. Additionally, a high BMI independently influenced the prevalence of SD3 statistically.

## DISCUSSION

Sexuality, including sexual function, satisfaction, and/or family planning, is a major part of the quality of life for women of reproductive age. 21 Sexual dysfunction has major impacts on the quality of life of patients with MS. Improving the quality of life and functioning of these patients is a critical responsibility of MS healthcare professionals.

In the context of female sexuality, Basson (2021) proposed a circular model to provide a better depiction of sexual response in women. The proposed model defines the role of seduction, arousal, and emotional intimacy in triggering female sexual desire. An important aspect of Basson's circular model is its emphasis on the significance of the emotional context and relationships in respect of female sexual response. In contrast to earlier models that primarily focused on physical arousal, Basson's model recognizes that desire and motivation for sexual activity can arise from emotional and social factors, in addition to direct physical stimuli. In this cycle, there can be disruptions to the sexual response cycle, which may lead to sexual dissatisfaction or even sexual dysfunction. 22 These changes may be promoted by the presence of chronic diseases, use of medications such as birth control pills or antidepressants, the quality of sexual relationships, and social factors such as unemployment. 23

Estimates of the global prevalence of SD in women range from 30 to 67.7%. 24 25 26 In the present study, there were high SD rates in both groups, with no statistically significant difference between them using the FSFI tool. Likewise, no differences between the two groups were evident in respect of the scale domains. Thus, the rate of SD was not higher in patients with RRMS, nor were there differences in the scale domains in the underlying causes of SD in the groups assessed using the tool. Kołtuniuk et al. (2020) also reported a similar finding when using the same FSFI analysis tool. 27 The prevalence of SD detected in the present study is consistent with rates reported for the general population. Thus, although there was no statistically significant difference in the prevalence of SD between the RRMS and control groups, the rates of SD were high according to the FSFI. The instrument comprises questions assessing sexual response (desire, arousal and sexual satisfaction, lubrication, and orgasm), so the results reflect SD in the female population based on these components only. Compared with the circular model of sexual satisfaction outlined earlier, the FSFI does not probe other categories that are involved in sexual satisfaction such as intimacy, emotional satisfaction, and sexual stimulation. These aspects, when assessed, may increase or decrease this prevalence, as sexual response should not be the sole element evaluated when assessing sexual satisfaction. Moreover, the FSFI fails to identify the potentially different underlying causes for SD when comparing the RRMS patients with the control subjects.

In contrast, the MSISQ-19 demonstrated a higher prevalence of SD in patients with RRMS compared with that indicated by the FSFI in this population. The MSISQ-19 detected an SD rate of 56.4%, with a statistically significant difference relative to the rate determined by the FSFI. These findings are consistent with the results of Mohammadi et al. (2014), whereas Carotenuto et al. (2021) found a positive correlation between the two instruments in the MS population studied. 18 28 This high prevalence mirrors the results of another recent study by our group, which found an incidence of SD of 63.8% in female patients using the MSISQ-19. 29

The most common problems identified on the scale domains were those stemming from the sequela of the disease (manifesting as SD2). Thus, because the MSISQ-19 contains specific questions for people with MS, it was able to detect a higher percentage of SD in patients with RRMS compared with the FSFI tool. The MSISQ-19 has demonstrated excellent sensitivity, becoming the main clinical screening tool for SD in people diagnosed with MS. 17 30 31 These findings show the superiority of the MISISQ-19 over the FSFI in females with MS, thus suggesting that the instrument is a more appropriate screening tool for SD in the MS population.

Such screening is vital since sexuality is an under-investigated issue in routine consultations and is not a complaint that is frequently raised spontaneously by patients. Moreover, some professionals find it difficult to talk about sexual function, which may be a barrier to the assessment of the sexual health needs of their patients with MS. Thus, the use of validated questionnaires such as the MSISQ-19 may provide a framework for discussion and yield specific, actionable information about patients' SD that providers can use to provide targeted treatments and interventions.

Another pertinent finding of the current study was the high rate of anxiety and depressive symptoms identified in both groups studied. The high prevalence of anxiety and depression in the population with MS has been extensively described in the literature, 32 33 34 as has the association of these comorbidities with SD. 35 36 However, no significant difference in the prevalence of these disorders was detected between the patient and control groups in the present study despite the use of two tools assessing mood (HADS and BAI). So, this means that although anxiety and depression may be linked to SD, they cannot be singled out as factors that affect people with MS.

An analysis of independent factors related to the population with RRMS diagnosed with SD found that a higher BMI was associated with SD3, which is in agreement with prior studies. This may be related to the physical sequelae of being overweight as well as the increased incidence of depression and anxiety in obese patients. 37 This underscores the importance of active screening and treatment anxiety and depressive symptoms and higher BMI in people living with MS, which may help to reduce the prevalence of SD.

Surprisingly, we found that shorter disease duration was correlated with a higher prevalence of SD. The diagnosis of MS is a major life stressor for patients, and the physical and neurological sequelae of the first episode of MS can have a major impact on the sex lives of patients early in the disease course. Thus, it is vital that professionals involved in the treatment of MS discuss sexual health early and often in the disease course. We recommend addressing the subject of SD in the first sessions following the diagnosis of the disease, as soon as a bond of trust has been established between the patient and the care team.

The present study has some limitations which should be noted. First, only patients diagnosed with RRMS with mean scores of 2 or more on the EDSS were assessed, explained by the age cut-off of 45 years (probably not including patients with progressive phases of the disease), and by the consecutive recruitment of patients. Third, all patients were included, irrespective of their sexual orientation, with no changes to the tools used to assess specific SD. The scales employed in the present study have not been adapted for homosexual relationships; thus, future studies should investigate the effectiveness of these tools in this specific group.

In conclusion, the MSISQ-19 should ideally be used to assess SD in this patient population, and we recommend that sexual health should be a routine topic in the care of MS patients, as the physical, neurological, and psychosocial factors related to the disease are closely linked to sexual function and satisfaction. Regularly addressing sexual health in MS patients can help improve their quality of life and overall well-being.
